# Annotations

**Published:** 1907-02-02

**Authors:** 


### ANNOTATIONS.

### Street Noises in London.

THE deputation representing the Betterment of London Association, which waited on the Home Secretary on January 24 in reference to the noise and other disagreeable features associated with motor omnibuses and traction engines, represented, no doubt, a considerable amount of personal discontent and even actual suffering. But it may be questioned whether their case was wisely presented, and still more whether the speakers who summitted it exhibited a reasonable readiness to acknowledge the actualities of the position. Exaggerated phrases and denunciation suggest a somewhat narrow and personal view, which, whilst it has its due claim, cannot be allowed to dominate the situation. Whatever their faults, it cannot be denied that the motor omnibuses are proving an enormous convenience and benefit to large numbers of the hard-working section of the community; and to ask that all this shall be pushed to one side because the nerves of certain sensitive persons on the lines of route are upset by the traffic, argues a defective mental perspective. That the annoyance should be kept at the lowest possible level all will agree. Hence it is only right that in the selection of the lines of the motor-bus traffic regard should be paid to the character of the various neighbourhoods, and that quiet residential districts should, as far as possible, be preserved from disturbance. But even here it is the public convenience which in the last resort has to be considered. Again, it is eminently desirable that the noise created by public vehicles should be kept at a minimum. The deputation above referred to seemed to think that the Commissioner of Police was careless in this direction. But this is not in harmony with common experience. The noise and fumes arising from the motor-buses have in recent months distinctly declined, and we shall all be glad to see them at a still lower level. But to ask for the suppression of these buses in the interests of private individuals is a vain dream, and we are glad to see that the Home Secretary in his reply put the position in very plain terms.

### The Necessity for Special Hospitals.

THE people of Salford and Manchester have shown marked liberality, in recent years, towards their medical charities. The latest instance is the subscription of £33,000 towards the £38,000 required to defray the cost of the new special hospital for Skin Diseases, in Quay Street, Deansgate. Sir Thomas Barlow, at the opening ceremony, expressed satisfaction that this hospital has been placed in the centre of a poor population, which pre-eminently needed its help. Voluntary hospitals, if they are to be of real service to the people, must go to the poor, rather than compel the poor to come to them—that is, they must select a site near the homes of those who constitute fit objects for free hospital relief. Skin diseases do not appeal, perhaps, to the sentiment of humanity, in the same way that many other diseases do. Yet skin diseases in many forms, though not of a crippling or vital nature, may markedly interfere with the joy of life, and prevent the sufferers from following many occupations. Sir Thomas Barlow, had reason on his side, when he expressed satisfaction at municipalities taking pride in their cities, as shown by the exhibition of energy in the removal of everything that was either ugly or displeasing, and in securing fine boulevards and attractive public buildings, so that our great towns may be made as beautiful as possible. But there are other and more pressing claims which the poor town-dweller has upon the public and the authorities. Because it is right to beautify towns and make them as healthy and attractive as possible, it is even more essential to do what is possible to safeguard the human form divine, and to remove from the poorer residents every sense of bitterness and repining at their lot, by supporting a special hospital for skin diseases, which must, in the ordinary course of its work, do much to conserve that beauty in individuals, which is one of the joys of life. Probably, there are few diseases, which are more obstinate and intractable, than certain forms of skin disease. The longer adequate treatment is withheld, the greater is the discomfort and disability which result. We therefore congratulate the people of Manchester, upon their liberality and wisdom, in having built a modern, up to date, skin hospital. It should prove of the greatest advantage directly to the poor, and indirectly to the whole of the residents in this great commercial centre.

### Leprosy in Cape Colony.

THE address on this subject recently given by Dr. Sutherland Black at the Medical Graduates' College and Polyclinic, has the special interest and value which are attached to the statements of one who has had long, intimate, and practical experience of the disease in question. After seven years' experience at Robben Island, where some 1,200 lepers are segregated, Dr. Black expresses his unhesitating conviction that the disease is communicable from one person to another, and he has found no reason to believe in the agency of any intermediate host. It is upon this view of the contagiousness of the disease that the Leprosy Repression Act and the isolation of the lepers at Robben Island are based, and, moreover, both the doctrine and the practice are generally accepted by the medical profession in South Africa. Notwithstanding all this, Dr. Black admitted that there were indications of a decided extension of the disease in Cape Colony, and that probably there are as many lepers at large as there are confined in the leper asylums. He also suggested that certain forms of the disease are only infectious during the existence of a brief and transient nasal ulceration. In reference to treatment, Dr. Black stated that he had found some good from creasote, but had obtained the best results from chaulmoogra oil in large doses. In spite, however, of the definite views on certain points, the lecturer recognised that even yet a great deal of uncertainty attends our knowledge of the pathology and management of leprosy, and he urged that a special commission should be sent out to South Africa to investigate the whole question.

